# An overview of city analytics

**DOI:** 10.1098/rsos.161063

**Published:** 2017-02-01

**Authors:** Desmond J. Higham, Michael Batty, Luís M. A. Bettencourt, Danica Vukadinović Greetham, Peter Grindrod

**Affiliations:** 1 Department of Mathematics and Statistics, University of Strathclyde, Glasgow, UK; 2 Centre for Advanced Spatial Analysis, University College London, London, UK; 3 Santa Fe Institute, Santa Fe, NM, USA; 4 Department of Mathematics and Statistics, University of Reading, Reading, UK; 5 Mathematical Institute, University of Oxford, Oxford, UK

**Keywords:** clustering, crowdsourcing, inference, modelling, networks, scaling

## Abstract

We introduce the 14 articles in the Royal Society Open Science themed issue on City Analytics. To provide a high-level, strategic, overview, we summarize the topics addressed and the analytical tools deployed. We then give a more detailed account of the individual contributions. Our overall aims are (i) to highlight exciting advances in this emerging, interdisciplinary field, (ii) to encourage further activity and (iii) to emphasize the variety of new, public-domain, datasets that are available to researchers.

## 1. Background

More than half of us live or work in a city. This proportion is growing rapidly and by the end of this century most of the world’s population will be urbanized. Most human interaction, energy consumption, waste generation, innovation, entertainment and education takes place in cities. City life generates data streams around, for example, online social media, telecommunications, geolocation, crime, health, transport, air quality, energy, utilities, weather, CCTV, wi-fi usage, retail footfall and satellite imaging. From the stakeholder side, there is a big external pull for these data streams to be fully and appropriately exploited. Within the research community, there is a corresponding internal push for cutting-edge models, algorithms and tools to be tested, customized and deployed, and where necessary, for new, high-quality advances in data science.

What is different about city analytics?

— It sits in a challenging, interdisciplinary space.
— It interacts with multiple sectors (transport, energy, security, well-being, commerce, governance, environment and resilience) and key professions (architecture, engineering, policy-making and urban planning).
— It involves disparate new types of data, much of which is large-scale (CCTV, social media, city sensors, retail, utility and population censuses).
— It raises novel issues in terms of privacy and ethics.

Around the world, numerous City Lab style initiatives are being developed in order to push forward the research agenda. In the UK, government funds have recently supported, for example, the Smart City Demonstrator programme in Glasgow, the Future Cities Catapult in London and the Urban Science Centre for Doctoral Training at the University of Warwick. Moreover, the Alan Turing Institute, which is a national institute for the data sciences, has chosen Smart Cities as one of its six priority themes. There are other laboratories funded from various sources, such as the Centre for Advanced Spatial Analysis at University College London, the Institute for Future Cities at the University of Strathclyde, the Center for Urban Science and Progress at New York University, the ETH Future Cities Lab in Singapore, the SENSEable City Lab at MIT, and the Urban Center for Computation and Data at the University of Chicago/Argonne National Laboratory. Each of these laboratories has a different focus with respect to the interdisciplinary mix of sectors and professions noted above.

## 2. The themed issue

The call for papers for this themed issue of Royal Society Open Science on *City Analytics* was launched at a workshop in September 2015. The workshop, hosted by the Future Cities Catapult at the Urban Innovation Centre in London, UK, brought together a wide range of interested parties from academia, industry and government, and included contributions from Michael Batty (UCL), Fran Bennet (Mastadon C), Scott Cain (Future Cities Catapult), Ewen Gibb (Future Cities Catapult), John Gibson (Nesta), Peter Grindrod (University of Oxford), Des Higham (University of Strathclyde), Jeremy Morley (Ordnance Survey), Cathy Mulligan (Future Cities Catapult), Chris White (EPSRC) and Hyejin Youn (University of Oxford).

In the call for papers, we asked for contributions that

— develop and test novel mathematical models or novel computational tools to help us understand modern, urban, environments, and/or
— apply existing, state-of-the-art, mathematical models or computational tools in order to gain new insights about urban life.

We sought research articles produced by teams that include expertise in mathematics/statistics, physics or computer science, and we encouraged interdisciplinary collaboration across areas such as social science, geography, engineering, business, epidemiology, health informatics and human psychology. The themed issue was motivated by the emergence of new and open datasets that are driving novel research and offering opportunities and challenges in this highly interdisciplinary field. All published articles in the issue therefore contain illustrative results on realistic data streams that relate to city life. The journal kindly offered to waive page charges for these articles and to grant automatic gold standard open access.

The editors received a total of 23 submissions, all of which were carefully peer reviewed and edited. Upon acceptance, articles were immediately published online. In total, 14 articles appear in this themed issue, giving an acceptance rate of around 60%.

## 3. Summary information

Table 1 gives an at-a-glance overview of the contents of this issue. For each article, we have recorded the broad topic within city analytics where new insights have been obtained. We also list key computational tools used, and indicate circumstances where the authors found it appropriate to advance the state of the art in terms of introducing new algorithms or models. In this context, the phrase ‘model’ can be interpreted in either a statistical sense—as a putative relationship between observed variables—or in a mathematical sense—as a set of physically or empirically derived laws of motion (which may themselves incorporate randomness). The table also flags up which articles make use of newly captured, or newly curated, datasets. The journal’s Open Data policy requires ‘supporting data and information, including source code, to be made available prior to publication so that all results are reproducible … Unless there are strong extenuating circumstances …’ As noted in the table, 12 of the articles make all data available. The exceptions are 5, where similar data are available in the public domain, and 8, where the mobile phone data are proprietary and subject to privacy regulations. Although the articles focus largely on methods, some do suggest new and different ideas about how city systems work and it would be remiss not to think of these as contributing to novel theories about how cities develop and function.

**Table 1.** Summary of some key characteristics of the 14 articles in this themed issue. ABM, agent-based modelling; BIC, Bayesian information criterion; PCA, principal component analysis.

references	insights into	key techniques	new alg.	new model	new data	data available
Aiello *et al.*^1^	urban sound	PCA, soundwalks			√	√
Alessandretti *et al.*^2^	transport	networks, factorization	√			√
Arcaute *et al.*^3^	city boundaries	percolation, fractals				√
Charlton *et al.*^4^	social media	networks, ABM	√		√	√
Daggitt *et al.*^5^	urban growth	spectral clustering	√			
Grindrod & Lee 6	social media	clustering, random graphs	√	√	√	√
Leitão *et al.*^7^	scaling laws	likelihood, BIC	√	√		√
Lenormand *et al.*^8^	land use	clustering, Ripley K		√		
Lotero *et al.*^9^	mobility	networks, clustering	√	√	√	√
Pregnolato *et al.*^10^	flooding	networks, hydrodynamics				√
Seresinhe *et al.*^11^	art/economics	regression, bootstrapping			√	√
Tkachenko *et al.*^12^	flooding	PCA, eigencities				√
Ward *et al.*^13^	footfall	ABM, data assimilation		√		√
Williams & Musolesi 14	transport	networks, attacks	√	√	√	√

## 4. Further details

A key feature of the ‘Living Lab’ paradigm is that individuals generate data while going about their daily lives; for example, by posting social media messages in the public domain without being aware that this information might be used by data analysts. In this sense, the recorded observations are unbiased—although it must also be acknowledged that the sample population itself may represent a non-uniform selection from the whole. Such ‘crowdsourcing’ of our digital footprints has proved to be successful in many commercial settings and, when handled appropriately, can inform academic studies. In particular, many hypotheses from the social sciences that were impractical to verify in the pre-digital age may now be tested at scale. In this vein, Seresinhe *et al*. 11 address the question of whether there is a link between the presence of art and the economic condition of an urban neigbourhood. To this end, the authors count geographically located photographs that have been uploaded to Flickr and tagged with the word ‘art’, and correlate this information with relative changes in mean property prices. After correcting for possible clustering effects, the authors conclude that relative increase in house price is significantly associated with a higher proportion of art images. Picture tags were also exploited in 1, where Flickr uploads geotagged for Barcelona and London are compared with a specially compiled urban sound dictionary. This allows the authors to summarize city street segments in terms of their sound profiles. To add further insights, *soundwalks* are conducted, in which individuals are led along predefined routes and asked to summarize their responses to the sounds present. Creating such soundmaps on a city scale, by adding value to existing social media data, complements the traditional street plan with a novel and useful layer of information.

It is clearly of interest to improve our understanding of the way that individuals move to and from a city, and the way that they navigate within the city. Many of the articles in this themed issue deal with urban movement, either directly or indirectly. The authors in 13 consider a mathematical model for footfall along a high street in Leeds. Here an agent-based modelling (ABM) framework treats pedestrians as individual actors with their own behaviours and histories. The large volume of camera-generated data makes it feasible to use cutting-edge algorithms to initialize and calibrate the model, and to recalibrate dynamically as new data arrives. In this way, extracting the underlying ‘rules’ that individuals use to navigate a city can allow us, for example, to make quantitative predictions about future behaviour or response to perturbations. Alessandretti *et al*. 2 consider public transportation behaviour. Using metro, tram, bus and rail data associated with Paris, Toulouse, Nantes and Strasbourg, they develop new methodology to describe and analyse transportation networks, revealing hidden characteristics and quantifying efficiency. In 9, commuting patterns of individuals, as recorded from origin–destination surveys in the Columbian cities of Manizales and Medellín, are summarized and compared. In particular, because of the nature of the data, the authors are able to draw inferences about mobility patterns with respect to both the geographical locations of the origin/destination and the socio-economic status of the actors. Returning to social media data, in 5 location-based information from Foursquare is used to study urban growth across 100 major cities. By crowdsourcing data about individuals’ locations, the authors are able to study growth patterns, detect spatial correlations and surges, and quantify competition and cooperation between retail outlets.

Our urban landscape can be viewed at many scales, and any conclusions that we draw about the existence of patterns, including clusters, boundaries and fractures, must acknowledge the calibration of the ‘microscope’. To emphasize this issue, Arcaute *et al*. 3 treat road intersections as a proxy for urbanization, using Ordnance Survey data to represent the UK as a network with over 3 million nodes, and nearly 4 million edges. Applying percolation theory, they show that, depending on the level of hierarchy, Britain can be broken down into a variety of substructures, ranging from a north–south divide to the emergence of major cities, that generally relate to ideological, geographical and socio-economic divisions. They further show that fractal theory can be used to quantify the dimension of these structures. It is also noted that this approach gives a new framework for defining the boundary of a city—a concept that leads us into 7. If we agree on a definition of city boundaries, then we may compare population size, *x*, against some observation, *y*, such as the annual cinema attendance. Scatter plotting this data for a range of cities might tempt us to postulate that a *scaling law* exists: *y*=*x*^*β*^ for some ‘universal cinema parameter’, *β*. The authors show how to apply techniques of statistical inference to determine whether such a conclusion is valid, and in particular whether superlinear (*β*>1) or sublinear (*β*<1) behaviour is present. Their tests on 15 city datasets also emphasize that the conclusions typically depend strongly upon the assumptions that are fed into the model. The importance of scaling is also a key feature in 8. Here, the authors use geolocated phone records over the five most populated metropolitan regions of Spain—Madrid, Barcelona, Valencia, Seville and Bilbao—and perform clustering on correlations of activity. Unsupervised learning suggests four groups of land type, which correspond to residence, business, logistics/industry and nightlife. Via the Ripley K index and the entropy index, the patterns across different spatial scales are explored and seen to be consistent with a Schelling-like segregation model.

Two of the articles in this issue are concerned with flood risk. Pregnolato *et al*. 10 take a mechanistic approach, combining calibrated high-resolution flood and transport models to assess possible levels of disruption, and to quantify the benefits of flood risk management measures. Using historical data, a case study is presented for the city of Newcastle upon Tyne. In a more data-driven study, Tkachenko *et al*. 12 investigate links between geolocated ‘information seeking’ around flood events, via Google Analytics, and public-domain records of flood warnings and incidents. Patterns that emerge have a number of possible implications in the social and environmental sciences.

The majority of the articles in this issue draw on ideas from network science, and indeed three of them are completely embedded in this framework. Grindrod & Lee 6 use geolocated reciprocated Twitter mentions to build up a picture of the pairwise social interactions between inhabitants of 10 UK cities. They then break these networks down into modules, and test whether city A could be constructed out of modules from city B. This gives a novel technique for understanding and comparing communities within cities, and has implications for the use of social media campaigns and behavioural interventions. In 4, the transfer of mood through social networks is explored and modelled. The authors study the dynamic networks that Twitter users create through @-mentions over a period of several months, with the sentiments of the messages quantified. Analysis of established communities in those networks revealed that their average sentiment is relatively stable, and sudden changes can be traced to external events affecting the community. As the tools developed and tested here can be applied to any self-identifying or discovered communities, they have potential applications in, for example, energy, transport, tourism and policy-making. The work in 14 builds on classic network ideas; notably *centrality* measures that identify important or influential components. In particular, the concepts of closeness and betweenness are adapted and tested in a spatio-temporal setting. In this framework, taking into account both space and time helps us to understand vulnerability to disruption and attack. Results are given for several real-life networks, including examples built with data from the metro systems of London, Paris and New York, and from flight schedules in the USA.

## 5. Forward look

City analytics is inherently interdisciplinary, building on a wide base of foundational research contributions from a range of fields, including geography, the social sciences and architecture, as well as engineering, business and economics. It has been invigorated by the emergence of rich digital datasets that encompass a variety of aspects of city life. As the articles in this issue show, researchers in mathematics, statistics, computer science and related areas have something tangible to add to this mix. Working alongside domain experts, and exploiting the availability of new data streams, new technologies and committed stakeholders, we have the potential to validate theories about urban life, quantitatively compare competing hypotheses, draw inferences, make predictions and develop actionable insights.
